# Green synthesis of gold and silver nanoparticles using crude extract of *Aconitum violaceum* and evaluation of their antibacterial, antioxidant and photocatalytic activities

**DOI:** 10.3389/fbioe.2023.1320739

**Published:** 2024-01-10

**Authors:** Shahbaz Ahmad, Shujaat Ahmad, Qianqian Xu, Idrees Khan, Xiaoyu Cao, Ruimin Yang, Hai Yan

**Affiliations:** ^1^ School of Chemistry and Biological Engineering, University of Science and Technology Beijing, Beijing, China; ^2^ Department of Pharmacy, Shaheed Benazir Bhutto University Sheringal, Dir Upper, Khyber Pakhtunkhwa, Pakistan; ^3^ School of Chemistry and Chemical Engineering, Northwestern Polytechnical University, Xi’an, China

**Keywords:** green synthesis, *Aconitum violaceum* (AV), gold nanoparticles, silver nanoparticles, antibacterial, antioxidant, photocatalyst

## Abstract

Green synthesis of metal nanoparticles (NPs) has received extensive attention over other conventional approaches due to their non-toxic nature and more biocompatibility. Herein we report gold and silver NPs (AuNPs@AV and AgNPs@AV) prepared by employing a green approach using crude extract of *Aconitum violaceum* Jacquem. ex Stapf. The synthesized NPs were characterized using Scanning Electron Microscopy (SEM), Transmission Electron Microscopy (TEM), Energy Dispersive X-ray (EDX), X-ray Diffraction (XRD), UV/Visible spectroscopy, Fourier Transform Infrared (FTIR), X-ray Photoelectron Spectroscopy (XPS), and Zeta Potential. Morphological analysis showed spherical and triangular shapes of the NPs with average size of <100 nm. The AuNPs@AV and AgNPs@AV exhibited effective antibacterial activities, with minimum inhibitory concentrations (MICs) of 95 and 70 μg/mL against *Lactobacillus acidophilus* (*L. acidophilus*) and 90 and 65 μg/mL against *Escherichia coli* (*E. coli*), respectively. Strong antioxidant effect of AuNPs@AV and AgNPs@AV were reported against DPPH radical and PTIO within range of IC_50_ values; 161–80 μg/ml as compared to the standard (23–11 μg/mL) respectively. Moreover, the AuNPs@AV and AgNPs@AV showed efficient photocatalytic activity and degraded 89.88% and 93.7% methylene blue (MB) dye under UV light, respectively.

## 1 Introduction

Nanomaterial are a class of materials that are characterized by possessing at least one dimension less than 100 nm (Khan et al., 2019; Salem et al., 2022a). This categorization encompasses a range of structures, including nanowires, nanotubes, etc. Nanoparticles (having size in range of 100 nm) have received significant attention due to the unique physical and chemical properties that arise primarily from their dimensions. These properties make them suitable in a broad array of applications (Hashem and Salem, 2022; Hashem et al., 2023) including gas sensors (Shin et al., 2023), lithium-ion batteries (Fang et al., 2023), dye-sensitized solar cells (Jin et al., 2023), adsorption (Debnath and Das, 2023), heterogeneous photodegradation (Mota et al., 2023), biosensing (Lei et al., 2023), and antibacterial substrates (lashin et al., 2023; Al-Askar et al., 2023). Additionally, NPs have shown a vast range of biological applications, including antibacterial, anticancer, and antioxidant properties (Singh et al., 2023). They represent a promising alternative to antibiotics because NPs can target most bacteria as well as fungi, and exhibit a broad spectrum of activity (Długosz et al., 2022).

Metal NPs are conventionally synthesized using various physical, chemical and biological methods (Ali et al., 2022; Hashem and El-Sayyad, 2023). Although NPs of chemical origin have fine quality, they suffer from high cost (derived from costly instrumentation and energy-intensive synthetic processes), generation of toxic by-products, low biocompatibility, and related environmental concerns (Khan et al., 2022). In contrast, green synthesis sidesteps many of these drawbacks, and has received increased attention from researchers (Manojkumar et al., 2023). This approach has several advantages over the traditional physical/chemical methods including simplicity, low cost, eco-friendliness, potential for large-scale production, reduced reagent requirements, and the production of more stable and biocompatible NPs (Aldeen et al., 2022). Green synthesis of NPs is based on fungi, plants and their extracts, and microorganisms such as yeast and bacteria (Salem et al., 2022b; Gur et al., 2022; Singh and Mijakovic, 2022), all of which are economically viable and are not harmful for the environment (Muddapur et al., 2022). Green-synthesized NPs endow remarkable improvements in biological applications (Hariharan et al., 2019). Among these, plant extracts are preferred over other approaches because of easy handling, safe production, one-step method, and large-scale facilitation without any hazardous by-products (Chandraker et al., 2022a; Rafique et al., 2022). Plants are also favored over other biological methods because they obviate the need for long times of fungal and bacterial culturing and preservation (Eltaweil et al., 2022). Significantly, plant extracts having medicinal properties can be applied to obtain NPs with enhanced bioactivities (Bordiwala, 2023). Plant extracts contain various bioactive molecules that have benefits in the reduction and stabilization of NPs (Karthik et al., 2022). Phytochemicals present in plant extracts can act as reducing, precipitating, and capping agents, thus exerting a significant effect in controlling particle size, shape, phase stability, and other key characteristics of NPs (Bannunah, 2023). Gold and silver nanoparticles are the subject of substantial research owing to their unique optical and electrical characteristics, biocompatibility, high surface area and stability (Ali et al., 2020a; Nadaf et al., 2022; Ali et al., 2023). Other metallic nanocomposites have valuable properties, but gold and silver nanoparticles are often used due to their biocompatibility and facile preparation (Ali et al., 2021a).

In the present work, a green approach was adopted for the synthesis of Ag and Au NPs (AuNPs@AV and AgNPs@AV) using crude extracts of *Aconitum violaceum* (AV). Ag NPs have attracted global attention for their antimicrobial activity (Debnath and Das, 2023) against bacteria, algae, fungi, and viruses (Ali et al., 2021a), and are widely utilized in this capacity in commercial products such as soap, food packaging, plastics, cosmetics, and textiles (Simon et al., 2022) owing to their unusual physicochemical properties (Chandraker et al., 2022b). Additionally, Ag NPs have attracted considerable scientific interest for their prospective uses (Sajjad et al., 2023) in biosensors (Selimoğlu et al., 2023), wound dressings (Sarviya et al., 2023), cancer treatments (Pushparaj et al., 2023), antioxidant activity (Lemus-de la Cruz et al., 2023), and antimicrobial agents (Balachandar et al., 2022; Nadaf et al., 2022) Gold NPs (Au NPs) are of significant interest due to their tunable and highly-localized surface plasmon resonance (Philip, 2009; Faid et al., 2023), with potential applications in biomedical science including drug delivery (Sarkis et al., 2023), magnetic resonance imaging (Kharey et al., 2023), and biosensors (Bisht et al., 2023). They have also shown (Selimoğlu et al., 2023) antioxidant (Li et al., 2023), anticancer (Abdulateef et al., 2023), and antimicrobial activity (Shirzadi-Ahodashti et al., 2023), leading to their use in wound dressings (Chen et al., 2023). AV is an ethnomedicinally important plant of the Himalayan region, where it is commonly used as a remedy for snake bites and severe pains (Sabir et al., 2016; Hadi et al., 2022). It has tremendous therapeutic effects owing to the presence of several alkaloids (aconitine and indaconitine) (Sabir et al., 2016), flavonoids (quercetin) (Yadav and Verma, 2010), benzoic acid, and other classes of natural products like steroids, glycosides, and anthraquinones (Miana et al., 1971). The plant is an inhibitor of cholinesterase enzyme (Khan et al., 2021) and has been demonstrated to inhibit the growth of various human cancerous cell lines (Acqua et al., 2008). AV extracts contain potent bactericidal constituents (Khan et al., 2021). The hypothesis for this study was to prepare (via green synthesis) gold and silver nanoparticles from AV extract with distinct physicochemical and biological features.

Owing to the immense importance of eco-friendly and sustainability of plant medicated metal nanoparticle preparation, the current study was aimed for silver/gold ions reduction into nano scale material using *A. violaceum* plant extract. The resulting nanoparticles were characterized properly using latest techniques while also evaluated for their selective antibacterial activities against *E. coli* (*Escherichia coli*) and *L. acidophilus* (*Lactobacillus acidophilus*) bacterial strains, antioxidant activities against DPPH and PTIO, as well as their photocatalytic potential in degradation of methylene blue dye.

## 2 Experimental work

### 2.1 Materials

Silver nitrate (AgNO_3_) and chloroauric acid (HAuCl_4_) were obtained from Sigma Aldrich, while glycerol is obtained from Beijing InnoChem Science & Technology Co., Ltd. Anhydrous sodium carbonate and magnesium sulfate anhydrous were received from Macklin. Potassium dihydrogen phosphate, calcium chloride dihydrate and Tween 80 were obtained from BRM Chemicals, while calcium chloride and ammonium ferric citrate were purchased from Sinopharm chemical reagent Co., Ltd. Malt dip powder and ammonium citrate tribasic were received from Shanghai yuanye Bio-Technology Co., Ltd. (Shanghai, China). All chemicals obtained were used without any further purification processes. The whole plant *Aconitum violeceum* was collected from Kumrat, Dir district, Pakistan. The plant was identified by Prof. Dr. Ali Hazrat, a Plant taxonomist, at the University of Malakand, Dir, Pakistan.

### 2.2 Preparation of plant extract and phytochemical tests

The plant *A. violeceum* was througly washed with tap water to remove any debris and shade-dried for 10 days. The dried biomass was ground to fine powder through a blender. The powdered material (100 g) was suspended in methanol (5 L) in a closed glass container for 10 days at room temperature. The methanolic extract was filtered and concentrated on a rotary evaporator to obtain methanolic crude extract (7.5 g) and stored at 4°C in a sealed container. The phytochemical tests to detect various classes of natural products were conducted for the crude extract through the procedures mentioned in literature (Bisht et al., 2023).

### 2.3 Green synthesis of AuNPs@AV and AgNPs@AV

To synthesize AuNPs@AV and AgNPs@AV, a standard green synthesis approach was employed with minor adaptations (Ali et al., 2021b). 5 mL of HAuCl_4_ salt (0.001 M in deionized water (DW)) was introduced into a beaker under continuous stirring for 5 min. This was followed by the addition of 5 mL of AV crude extract (20 mg/mL in DW), with pH adjusted to approximately 9 using pH meter. The reaction mixture was allowed to stir for 50 min, followed by centrifugation (6,000 rpm for 15 min) and three washing cycles. The effect of pH on the synthesis of NPs was studied by performing the reaction in different pH media. For the synthesis of AgNPs@AV, a parallel protocol was pursued involving the reaction of 5 mL of AgNO_3_ with 5 mL of AV (20 mg/mL DW) for 40 min with pH adjusted to 8.

### 2.4 Characterization of AuNPs@AV and AgNPs@AV

The green fabricated AuNPs@AV and AgNPs@AV were characterized by XRD analysis (D/MAX-RB X-ray diffractometer from Rigaku, Japan), employing a Cu Kα source (*λ* = 1.5418 Å) operating at 40 kV and 30 mA. The FT-IR spectrum was acquired using a Nicolet iS50 spectrometer from Thermo Scientific, United States. The morphology was analyzed using scanning electron microscopy (S-4800 microscope from Hitachi, Japan) operating at 20 keV. To analyze the UV-visible spectra, a UV-Vis spectrophotometer equipped with an integrating sphere (T9s; Persee, China) was employed, in which the blank reference was BaSO_4_. Additionally, TEM and HR-TEM imaging were obtained through a TEM apparatus (F-20, FEI, United States), with an acceleration voltage established at 200 kV. The XPS characterization was carried out utilizing an X-ray photoelectron spectrometer (ESCALAB 250Xi; Thermo, United States) employing Al Kα radiation.

### 2.5 Antibacterial assay

#### 2.5.1 Bacterial strains

*Escherichia coli* (ATCC 15224) and *L. acidophilus* (ATCC 4356) bacterial strains were selected as research samples for antibacterial evaluation. *E. coli* was cultivated on LB agar at a temperature of 37°C, while *L. acidophilus* stock cultures were cultured under the same condition but in a modified medium: 1 L is constituted by sucrose (8 g), glycerol (1 g), soybean meal (10 g), yeast extract (5 g), beef extract (5 g), ammonium citrate tribasic (2 g), malt dip powder (1 g), magnesium sulfate anhydrous (0.5 g), anhydrous sodium carbonate, (1.2 g), potassium dihydrogen phosphate (1 g), calcium chloride dihydrate (0.1 g), Tween 80 (1 g), calcium chloride (0.01 g), manganese chloride (0.005 g), ammonium ferric citrate (0.005 g). Prior to conducting the antibacterial assessments, both *E. coli* and *L. acidophilus* were sub-cultured onto fresh and appropriate agar plates for a duration of 24 h.

#### 2.5.2 Determination of MICs

*E. coli* and *L. acidophilus* were incubated for a night at 37°C, leading to bacterial cell count of 10^5^ CFU/mL. The minimum inhibitory concentrations (MICs) of the AV extract and their corresponding AuNPs@AV and AgNPs@AV were determined using the standard broth dilution approach (Ali et al., 2020b). A series of two-fold sequential dilutions with concentrations from 300 μg to 5 μg were prepared for AV extract, AuNPs@AV, and AgNPs@AV in Muller Hinton broth (Oxoid, UK). Inocula of *E. coli* and *L. acidophilus* containing 5 × 10^5^ CFU/mL cells were added to each dilution. Two controls were employed in the experimental setup: a positive control consisting of bacterial cells in growth medium and a negative control containing solely the growth medium. MICs of the AV extract, AuNPs@AV and AgNPs@AV were calculated as the minimum concentration of the tested samples that effectively inhibited bacterial growth.

### 2.6 Antioxidant activity of AuNPs@AV and AgNPs@AV

#### 2.6.1 DPPH radical scavenging activity

The assessment of 1,1-diphenyl-2-picrylhydrazyl (DPPH) activity of AuNPs@AV and AgNPs@AV was conducted following the reported approach with minor adjustments (Sreelekha et al., 2021). A solution of 1 mM DPPH was prepared in ethanol and combined with varying concentrations (0, 12.5, 25, 50, 100, and 200 μg/mL) of the green synthesized AuNPs@AV and AgNPs@AV. The mixture was thoroughly vortexed, and subsequently incubated at room temperature in the absence of light for 30 min to enable the NPs to interact with and scavenge DPPH radicals. To ensure the reproducibility of the results, the assay was performed in triplicate. The absorbance of each solution was assessed at 517 nm using a microplate reader, and the percentage of radical scavenging was computed for each concentration using the subsequent formula. A lower absorbance value indicates higher free radical activity within the reaction mixture. The radical scavenging activity (%) was calculated using Eq. 1.

$$RSA\;\left(\%\right)=\frac{Abs\;of\;Control-Abs\;of\;Sample}{Abs\;of\;Control}\times 100$$ (1)

#### 2.6.2 PTIO radical scavenging activity

The PTIO (2-Phenyl-4,4,5,5-Tetramethylimidazoline-1-Oxyl 3-Oxide) assay was conducted following a previously established methodology with slight adjustments (Li, 2017). Varying concentrations (0, 12.5, 25, 50, 100, and 200 μg/mL) of AuNPs@AV and AgNPs@AV were dispersed in deionized water. 200 μL of each concentration was added to 800 μL of PTIO solution (1 mM), and the mixtures were well-vortexed. Thereafter they were placed in darkness to minimize light-induced degradation of PTIO radicals and incubated for 2 h in a water bath at room temperature. Subsequently, the absorbance of each solution at 557 nm was gauged against the negative control. The percentage of PTIO scavenging was computed using formula (Eq. 2):

$$RSA\;\left(\%\right)=\frac{Abs\;of\;Control-Abs\;of\;Sample}{Abs\;of\;Control}\times 100$$ (2)

To ensure the reliability of the outcomes, the experiment was conducted in triplicate.

#### 2.6.3 IC_50_ determination of AuNPs@AV and AgNPs@AV

Graphs were constructed to illustrate the percentage of scavenging for both DPPH and PTIO in relation to the concentration of AuNPs@AV and AgNPs@AV. The IC_50_ value, representing the concentration at which 50% scavenging is achieved, was ascertained through regression analysis. A lower IC_50_ value signifies a more potent capacity to neutralize DPPH and PTIO radicals.

### 2.7 Photocatalytic performance of AuNPs@AV and AgNPs@AV

Green synthesized AuNPs@AV and AgNPs@AV were utilized for the photocatalytic degradation of methylene blue (MB) dye in the aqueous medium. In a typical reaction, 25 mg of AuNPs@AV and AgNPs@AV were dispersed separately in 30 mL of MB solution (30 ppm) and kept in the dark for 30 min at 25°C to attain adsorption-desorption equilibrium. The suspension was then irradiated under UV light with stirring for 150 min. Samples were taken at 30 min intervals and the photocatalyst was removed by centrifugation. The photodegradation of MB dye was measured from its absorbance at 668 nm using a UV-Vis spectrophotometer. The % degradation of MB dye was calculated from the following formula (Eq. 3):

$$\text{Degradation }\left(\%\right)=\left(\frac{A_{0}-A}{A_{0}}\right)\times 100$$ (3)

Where *A*_*o*_ represents the initial absorbance of dye and *A* stands for dye absorbance after the reaction.

## 3 Results and discussion

### 3.1 Green synthesis of AuNPs@AV and AgNPs@AV

**FIGURE 1:**
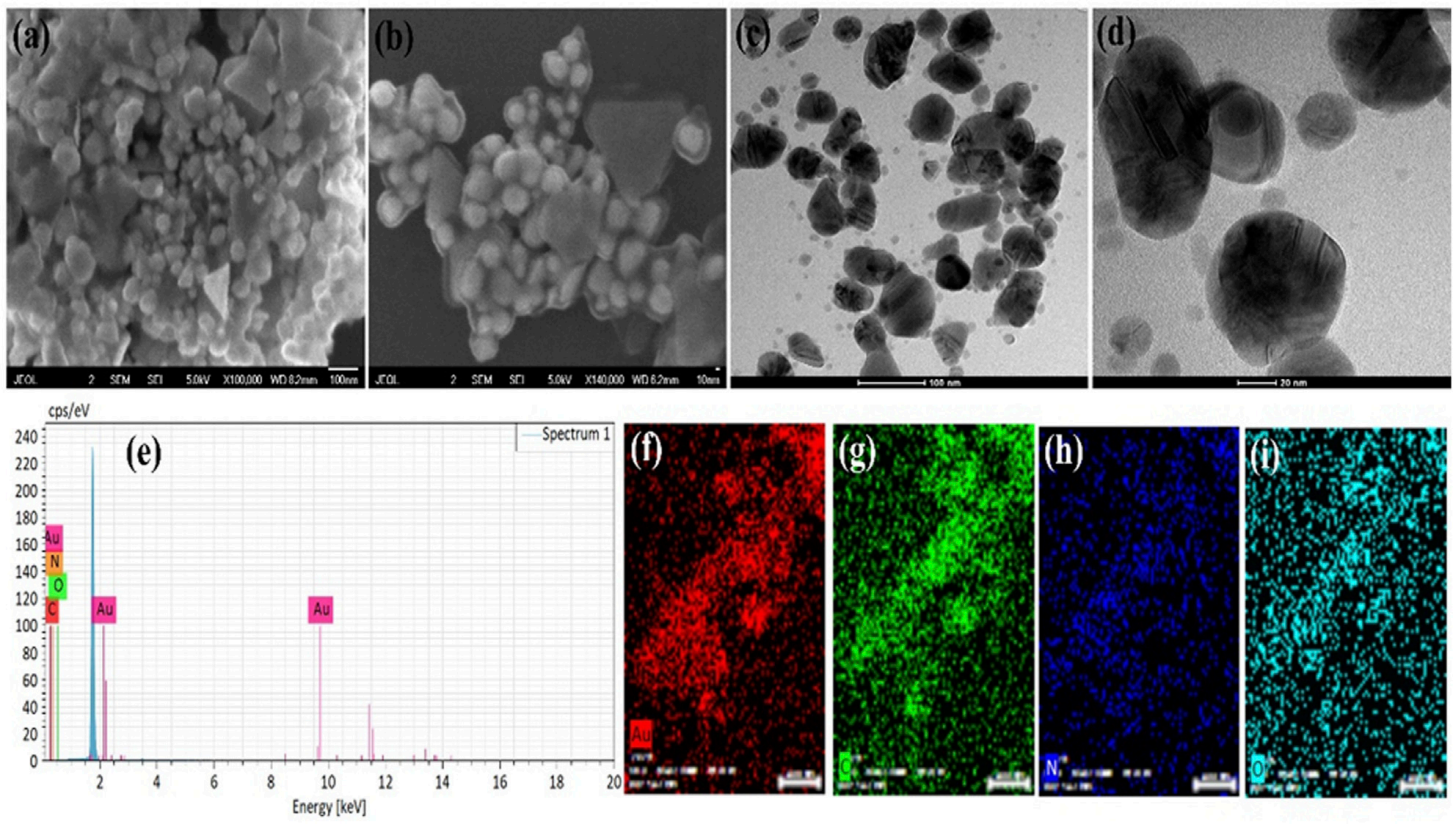
AuNPs@AV analysis via SEM imaging **(A,B)**, TEM imaging **(C,D)**, and EDX **(E)**. The constituent elements were spatially mapped [**(F)**, Au; **(G)**, Carbon; **(H)**, Nitrogen, **(I)**, Oxygen].

A straightforward and environmentally friendly method was utilized to prepare AuNPs@AV and AgNPs@AV. The resulting nanoparticles exhibited great stability, uniform size distribution, and demonstrated biological activity. The utilization of AV extract in the synthesis process functioned as both a stabilizing and reducing agent. The present methodology was based on previous instances of using plant extracts for the creation of silver and gold nanoparticles (Ali et al., 2020b; Ali et al., 2021b). The synthesis procedure entailed the reaction of AgNO_3_ and HAuCl_4_ with the AV extract under carefully optimized reaction conditions. This was supported by the noticeable changes in color observed in the reaction mixture, as depicted in Figure 1, which indicated the formation of AuNPs@AV and AgNPs@AV. Following the synthesis process, comprehensive characterizations of the AuNPs@AV and AgNPs@AV were conducted.

### 3.2 Characterization of AuNPs@AV and AgNPs@AV

#### 3.2.1 Morphology and elemental analysis

The morphology of the green synthesized AuNPs@AV and AgNPs@AV were examined through SEM and TEM analysis, while the elemental composition and distribution were studied via EDX analysis. Figures 1A, B shows the SEM images of AuNPs@AV at various magnifications, demonstrating spherical and triangular shapes. The NPs show some agglomeration which might be due to the presence of phytochemical in the AV extracts. These morphologies are further confirmed by the TEM images (Figure 1C, D) showing the deep investigations of the surface morphology. The SEM and TEM images also revealed that the size of the spherical AuNPs@AV is below 100 nm while the triangular size is near 100 nm. Figure 1E shows the EDX spectra of the AuNPs@AV with peaks for Au, C, N, and O. The presence of C, N, and O are due to the organic compounds present in plant extracts. Figures 1F–I represents the mapping results of Au NPs which also support the EDX spectrum results. Mapping results revealed that NPs contain phytochemicals which are responsible for biological activities.

**FIGURE 2:**
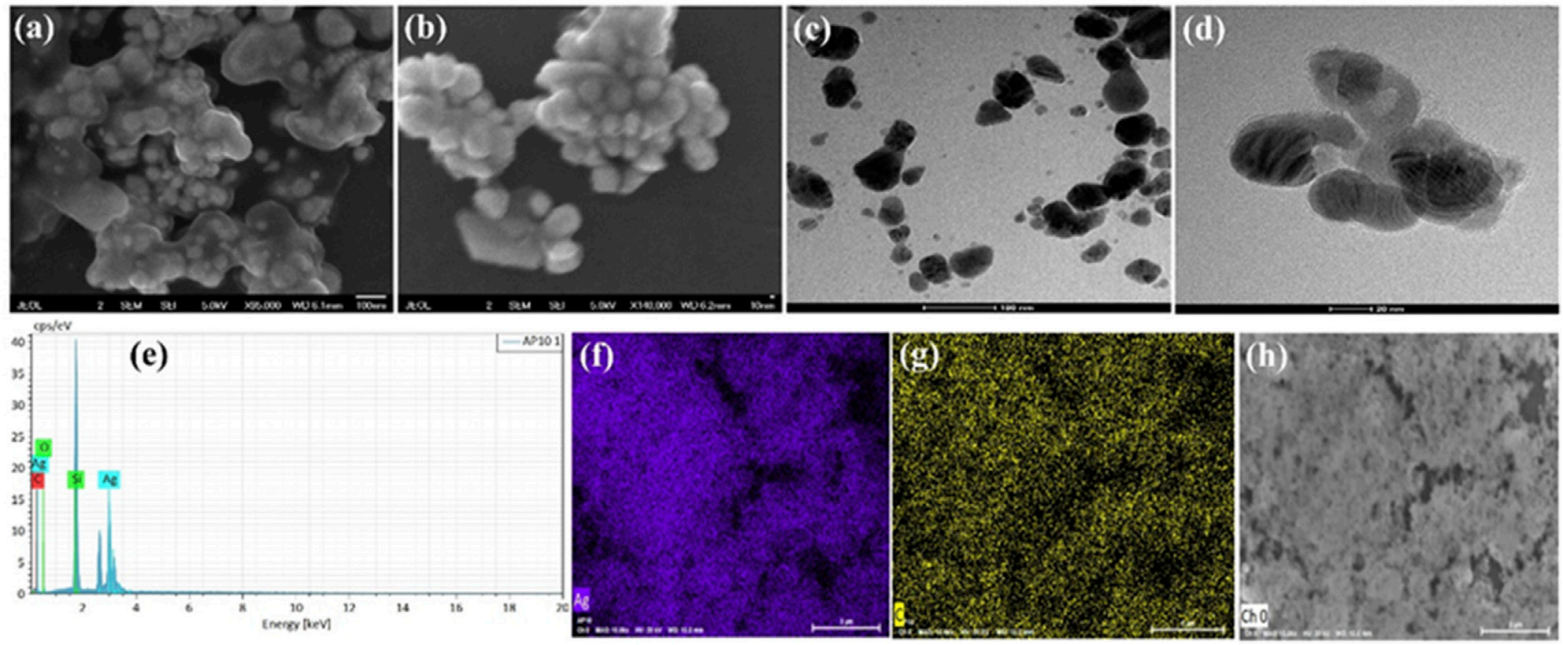
**(A,B)** SEM images **(C,D)** TEM images **(E)** EDX of AgNPs@AV **(F–H)** elemental mapping of the constituent elements of AgNPs@AV [**(F)**, Ag; **(G)**, Carbon; **(H)**, Oxygen].

Figures 2A, B demonstrates the SEM images of green synthesized AgNPs@AV at various magnifications. These revealed that AgNPs@AV have spherical geometry and were mostly in agglomerated form due to the presence of plant extracts. The morphology was further deeply investigated via TEM analysis and the results are represented in Figures 2C, D. Both morphological analyses confirmed the spherical morphology of AgNPs@AV with a size of less than 100 nm. The elemental composition was confirmed through EDX analysis (Figure 2E) representing peaks for Ag, C, O and Si. The presence of Si is due to the Si-grid used in elemental analysis. Figures 2F–H revealed the mapping results of Ag (red) occupying a larger area covered by oxygen (light blue) while a lower number of nitrogen (blue) and carbon (green) contents were also observed which also mean that organic compounds have bonded to the NPs and thus reduced them.

#### 3.2.2 XRD and FTIR analysis

**FIGURE 3:**
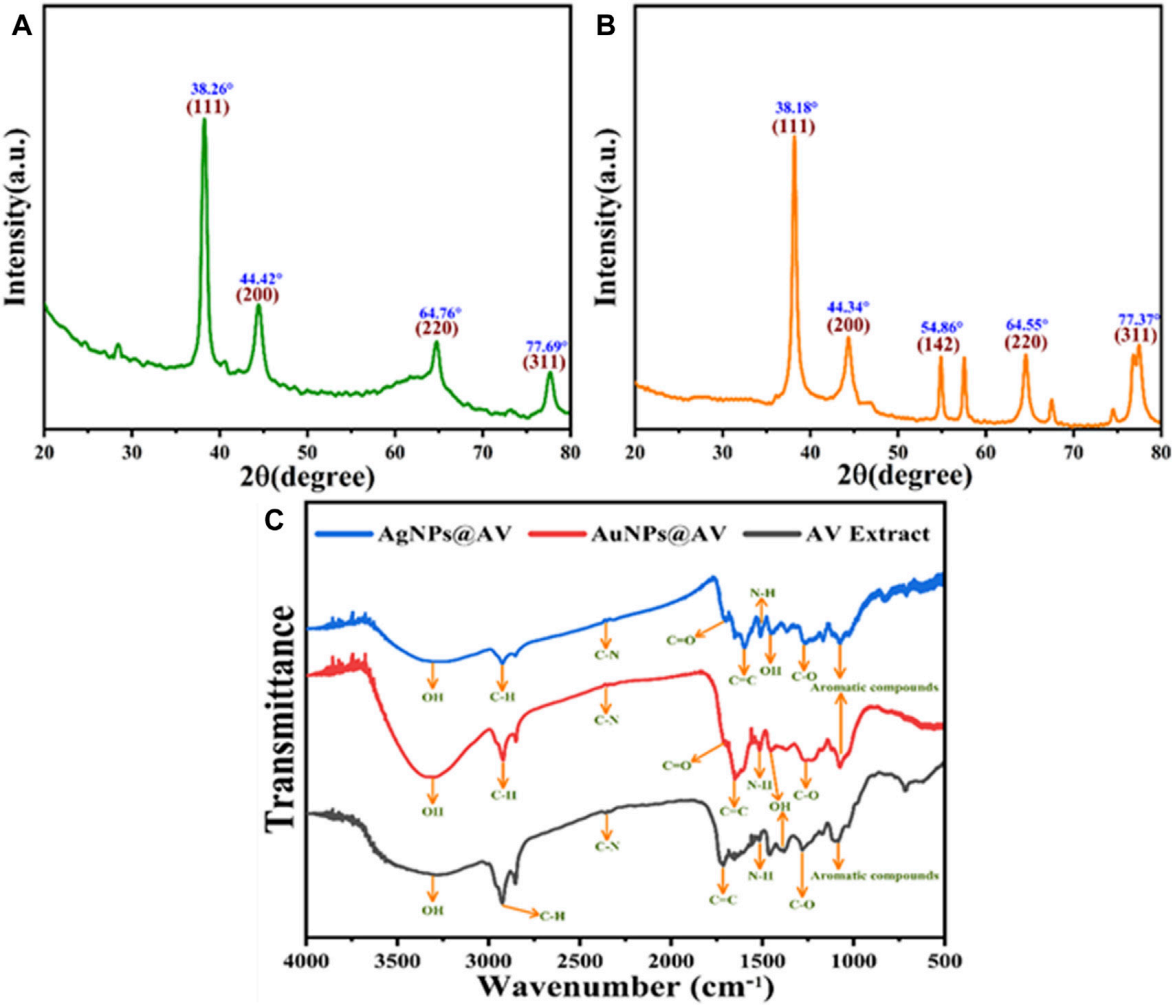
XRD pattern of the green synthesized **(A)** AuNPs@AV and **(B)** AgNPs@AV **(C)** FT-IR analysis of AgNPs@AV, AuNPs@ AV and AV extract.

An XRD pattern was acquired to determine the crystal facet alignment of the synthesized AuNPs@AV and AgNPs@AV (Figures 3A, B). AuNPs@AV (3a) featured significant peaks at 38.26°, 44.42°, 64.76°, and 77.69°, matching crystal planes (111), (200), (220), and (311), respectively. These findings strongly suggest the existence of a face-centered cubic nanoparticle structure within the produced AuNPs@AV. Similarly, the XRD patterns of AgNPs@AV (3b) featured peaks at 38.18°, 44.34°, and 77.73°, which can be attributed to the (111), (200), and (311) planes (Chandraker et al., 2022c; Deepa et al., 2023). FTIR spectroscopy analysis was conducted to identify distinct functional groups in the AV extract, AuNPs@AV and AgNPs@AV, as illustrated in Figure 3C. The FTIR spectrum of the AV extract exhibited noteworthy features, including a characteristic peak at 3,346 cm^-1^ attributed to the O-H stretching of phenols and alcohols. Additionally, the presence of tertiary amides was indicated by a peak at 1720 cm^-1^, corresponding to C=O stretching vibration, while an aromatic ether stretching of C-O was represented by a band at 1,265 cm^−1^, as depicted in Figure 3C. The FTIR analysis of AuNPs@AV revealed prominent peaks at 3,305, 1704, and 1,282 cm^−1^, which are associated with the O-H stretching vibration of phenols and alcohols, the C=O stretching vibration of tertiary amides, and the C-OH stretching of primary alcohols. Conversely, in the case of AgNPs@AV, distinct bands were noted at 3,292, 1,692, and 1,243 cm^−1^, signifying the stretching vibration of O-H for phenols and alcohols, the C=O stretching vibration for tertiary amides, and primary alcohols. These findings collectively provide evidence for the successful reduction of silver and gold metals (Isaac et al., 2013; Khan et al., 2020).

#### 3.2.3 XPS analysis of AuNPs@AV and AgNPs@AV

**FIGURE 4:**
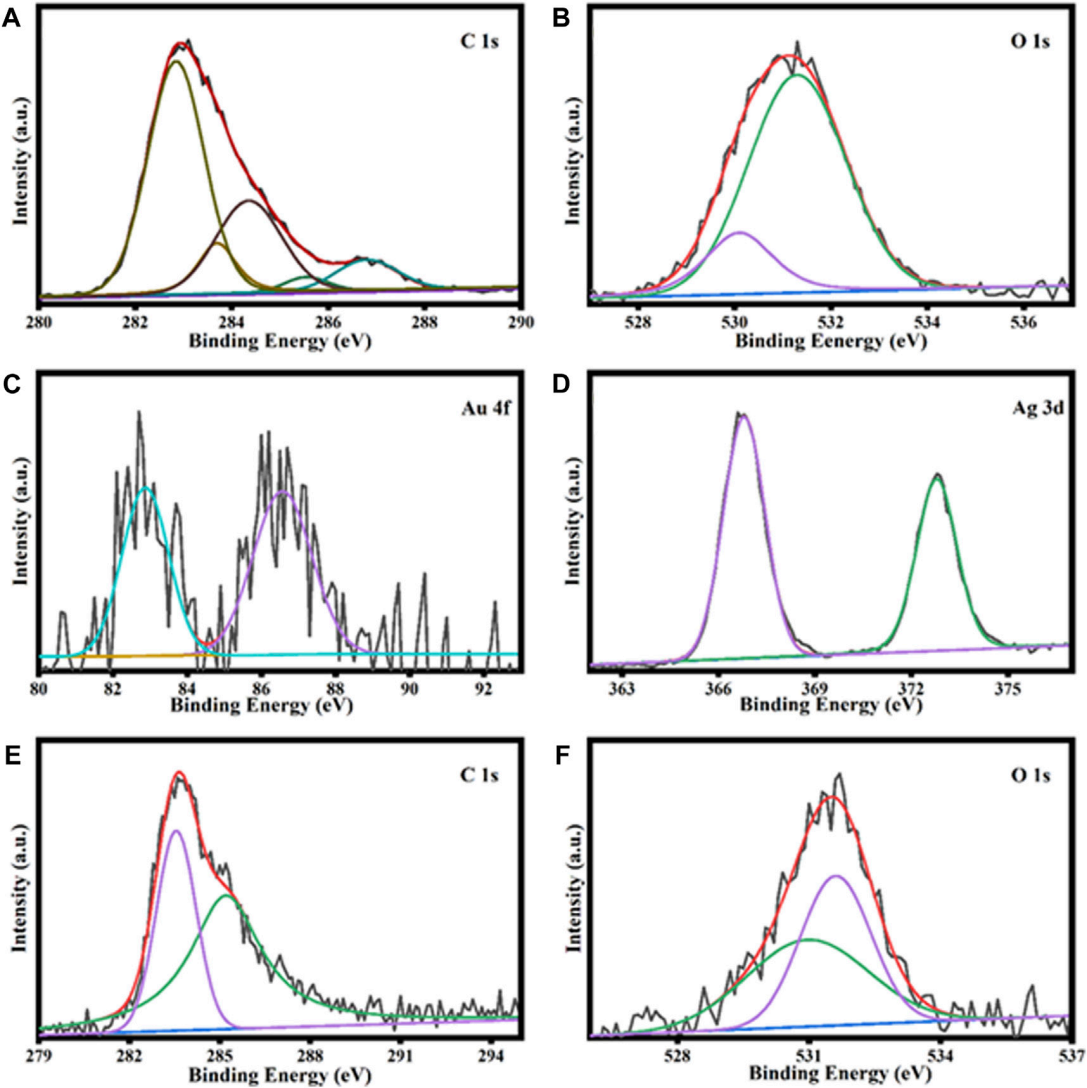
XPS analysis of AuNPs@AV representing carbon **(A)**, oxygen **(B)**, and gold **(C)**. Analysis of AgNPs@AV demonstrates silver **(D)**, carbon **(E)**, and oxygen **(F)**.

XPS was utilized to scrutinize the elemental constituents and bonding characteristics of the AuNPs@AV and AgNPs@AV as shown in (Figure 4). The results demonstrate signals corresponding to oxygen 1s, carbon 1s, and gold 4f, with individual peaks evident around 532, 284, and 85 eV, respectively. The C1s spectrum of AuNPs@AV in Figure 4A reveals characteristic peaks related to diverse carbon bonds such as C=O, C–O, C–C, and C=C. These peaks manifest at energies of 286.9, 286.1, 284.5/287.0, 286.3, and 284.7 eV, respectively. Figure 4B reveals the presence of peaks corresponding to Oxygen 1s at around 530.5 and 532 eV, respectively. Additionally, the binding energies associated with the 4f5/2 spin-orbitals in AuNPs@AV were discerned at 87.2 and 87 eV. Similarly, the peaks attributed to the 4f7/2 spin-orbital are evident at approximately 82.9 and 83.0 eV in Figure 4C (Xue et al., 2015; Rodríguez-León et al., 2019). XPS analysis of AgNPs@AV is given in Figures 4D–F. The Ag 3 days region exhibits a distinctive pattern, featuring two peaks situated at 373.5 and 367.4 eV. These peaks stem from the spin-orbital splitting attributed to Ag3d5/2 core levels, as shown in Figure 4D. Furthermore, Figure 4E shows the C (1s) spectrum, with intensities recorded at 284 and 285.7 eV that are indicative of C-O and C=O functionalities, respectively. The peaks identified at binding energies of 531.3 and 532.1 eV correspond to oxygen in AgNPs@AV, as demonstrated in Figure 4F. These observations signify the existence of a C=O and a C-O, respectively (Gurgul et al., 2011; Yang et al., 2013).

#### 3.2.4 UV study of AuNPs@AV and AgNPs@AV

**FIGURE 5:**
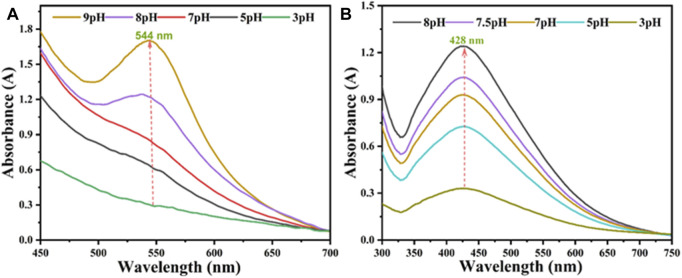
UV-Visible spectra of **(A)** AuNPs@AV **(B)** AgNPs@AV in different pH media.

The production of AV-fabricated AuNPs@AV and AgNPs@AV was assessed with UV spectroscopy to verify the best synthetic conditions. Ag or Au solutions (0.001 M) were separately reacted with the AV extract. Spectra were captured after completion of the reaction (30 min for AuNPs@AV and 70 min for AgNPs@AV) between 450 and 700 nm. Figure 5 illustrates the effects of varying pH levels at room temperature. In the case of AuNPs@AV (Figure 5A), the 544 nm absorption signifying nanoparticle production was negligible at pH 3, but rose progressively as the pH was increased to 9. Similarly, AgNPs@AV synthesis demonstrated continuous augmentation with increasing pH values, as demonstrated in Figure 5B. A distinct and well-defined peak was observed at 428 nm, which is very closed to the reported literature (Chandraker et al., 2019a; Hashem et al., 2022a; Kumar Chandraker et al., 2022). These outcomes further affirm the successful preparation of the NPs. Both AuNPs@AV and AgNPs@AV exhibited a preference for alkaline environments, with the reaction rate notably escalating as the pH shifted towards alkalinity because metal salt reduces effectively in the alkaline medium (Velmurugan et al., 2014; Biao et al., 2018).

**FIGURE 6:**
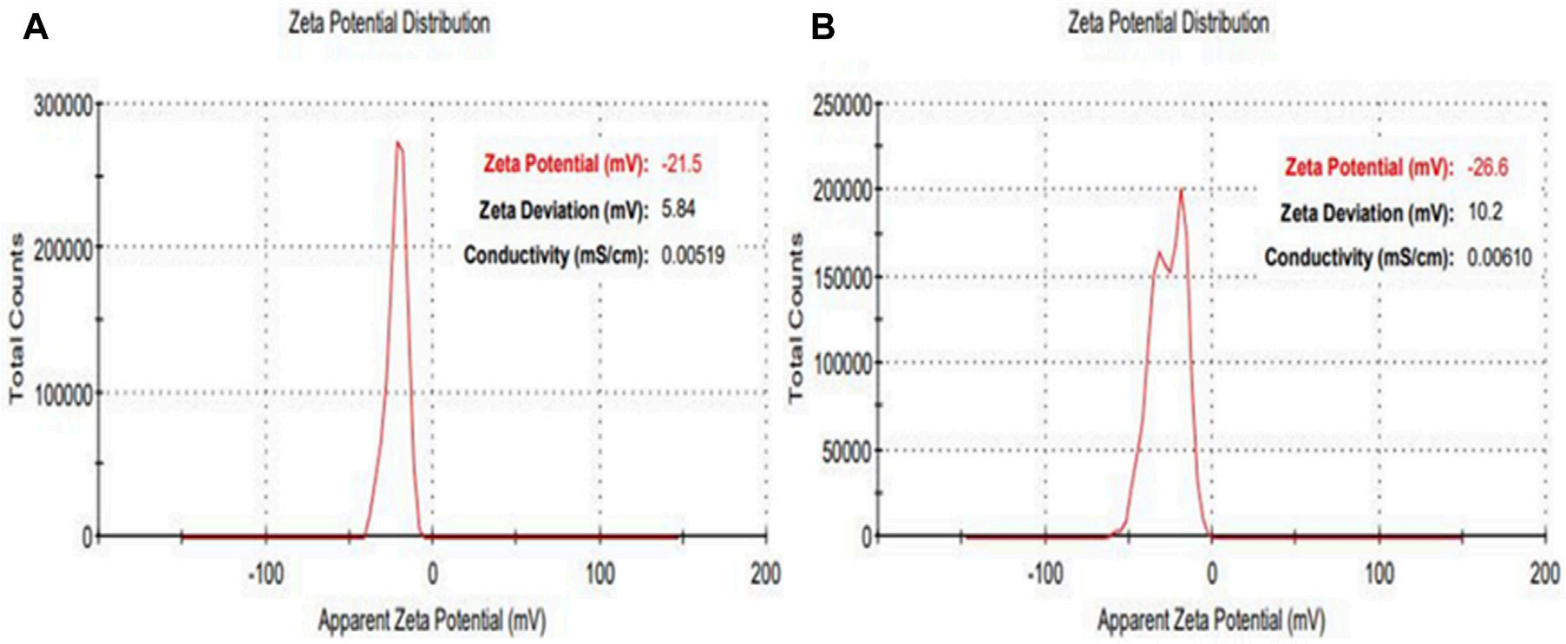
Zeta potential measurement of **(A)** AuNPs@AV **(B)** AgNPs@AV.

Additional characterization was conducted using Apparent Zeta potential analysis to assess both the surface charge and the stability of the synthesized NPs within a colloidal aqueous setting. As depicted in Figure 6, the zeta potential results portray the surface charge values for the as-prepared AuNPs@AV and AgNPs@AV. Figure 6A illustrates the stability of AuNPs@AV, indicating a surface charge of −21.5 mV. AgNPs@AV (Figure 6B) demonstrated a surface charge of −26.6 mV, indicating greater stability of the latter colloidal solution. The significant negative values suggest that the particles carry a substantial electric charge on their surface, leading to robust repulsion among the organically modified particles and effectively inhibiting aggregation in solution (Raj et al., 2018; Kim et al., 2021).

### 3.3 Antibacterial assay

NPs may harm bacteria and making it harder for bacteria to acquire resistance (Hashem et al., 2022b).

**FIGURE 7:**
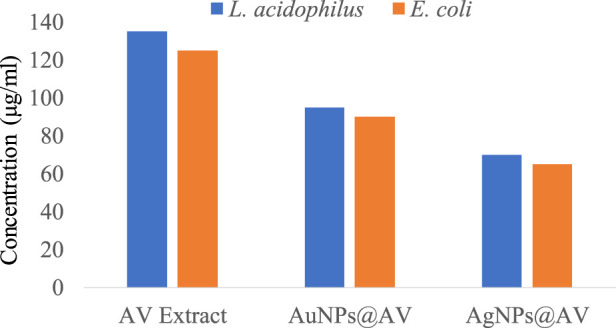
MICs of AV extract, AuNPs@AV, and AgNPs@AV against *Lactobacillus acidophilus* and *Escherichia coli.*

The antibacterial activities of the synthesized AuNPs@AV and AgNPs@AV were studied using a microtiter plate and broth dilution methods. The main advantage of NPs that bacteria cannot mutate their genes against NPs-mediated treatments (Chandraker and Kumar, 2022). In this study, *E. coli* (pathogenic bacteria) and *L. acidophilus* (beneficial bacteria) were tested to determine antibacterial selectivity of AuNPs@AV and AgNPs@AV. Hence, this research will assist future studies in balancing the protection of beneficial bacteria and the elimination of pathogens. *E. coli* is a Gram-negative bacterium that can cause foodborne infections and diseases, making a significant concern in food safety and public health (Yang et al., 2017; Alemu et al., 2023). *L. acidophilus* is a Gram-positive bacteria prevalent within the human gastrointestinal tract, known for its ability to make lactic acid and offer probiotic benefits that promote gut health (Wen et al., 2023). The concentrations of effective inhibition of pure AV extract, AuNPs@AV, and AgNPs@AV against *E. coli* and *L. acidophilus* are presented in Tables 1, 2. Figure 7 represents the MICs values of the AV extract, AuNPs@AV, and AgNPs@AV against the bacterial strains. The MICs of the AV extract were found to be 135 and 125 μg/mL against *L. acidophilus* and *E. coli*. The MICs of the AuNPs@AV were found to be 95 and 90 μg/mL against *L. acidophilus* and *E. coli*, respectively. Similarly, the MICs of the AgNPs@AV were found to be 70 and 65 μg/mL against *L. acidophilus* and *E. coli*, respectively. Notably, the NPs showed enhanced bactericidal activity relative to the AV extract due to the mutual antimicrobial effect of phytochemicals and NPs (Sabir et al., 2016). Among the NPs, AgNPs@AV established particularly strong antibacterial effectiveness, as expected given the known potent antimicrobial nature of Ag (Akter et al., 2020). AgNPs are used as a non-toxic inorganic antimicrobial agent against multidrug resistance bacteria owing to their biological activities (Chandraker et al., 2021a; Salem et al., 2022c), and well known against both Gram-positive and Gram-negative pathogenic bacteria (Huq and Akter, 2021). The well-defined uniform structure of AgNPs@AV contributed to their heightened efficiency, as the antibacterial activities of NPs depend on their morphology and size (Ali et al., 2020b). Previous research has also confirmed the biological activities of the AV extract (Sabir et al., 2016). Moreover, it was found that the AuNPs@AV and AgNPs@AV were more potent against *E. coli* as compared to *L. acidophilus*. This could be attributed to the difference in their cellular structures, as *E. coli* have comparatively thin peptidoglycan and protein layers which are vulnerable to AgNPs@AV (Chirumamilla et al., 2023).

**TABLE 1:** Percent inhibition (mm) of *Lactobacillus acidophilus* in response to increasing concentrations (5–200 μg/mL) of AV extract, AuNPs@AV, and AgNPs@AV.

Concentration (µg/mL)	AV extract	AuNPs@AV	AgNPs@AV
5	-	07 ± 0.2	09 ± 0.5
25	10 ± 0.1	15 ± 0.6	18 ± 0.8
50	19 ± 0.4	28 ± 0.8	33 ± 0.6
75	30 ± 0.8	40 ± 0.7	50 ± 0.2
100	42 ± 0.5	55 ± 0.4	58 ± 0.1
200	65 ± 0.3	75 ± 0.3	78 ± 0.4

**TABLE 2:** Percent inhibition (mm) of *Escherichia coli* in response to increasing concentrations (5–200 μg/mL) of AV extract, AuNPs@AV, and AgNPs@AV.

Concentration (µg/mL)	AV extract	AuNPs@AV	AgNPs@AV
5	-	08 ± 0.3	10 ± 0.1
25	12 ± 0.3	17 ± 0.3	20 ± 0.1
50	25 ± 0.2	32 ± 0.4	38 ± 0.5
75	32 ± 0.9	45 ± 0.5	55 ± 0.3
100	44 ± 0.7	57 ± 0.5	60 ± 0.2
200	68 ± 0.2	78 ± 0.3	80 ± 0.3

### 3.4 Antioxidant activities of AuNPs@AV and AgNPs@AV

**FIGURE 8:**
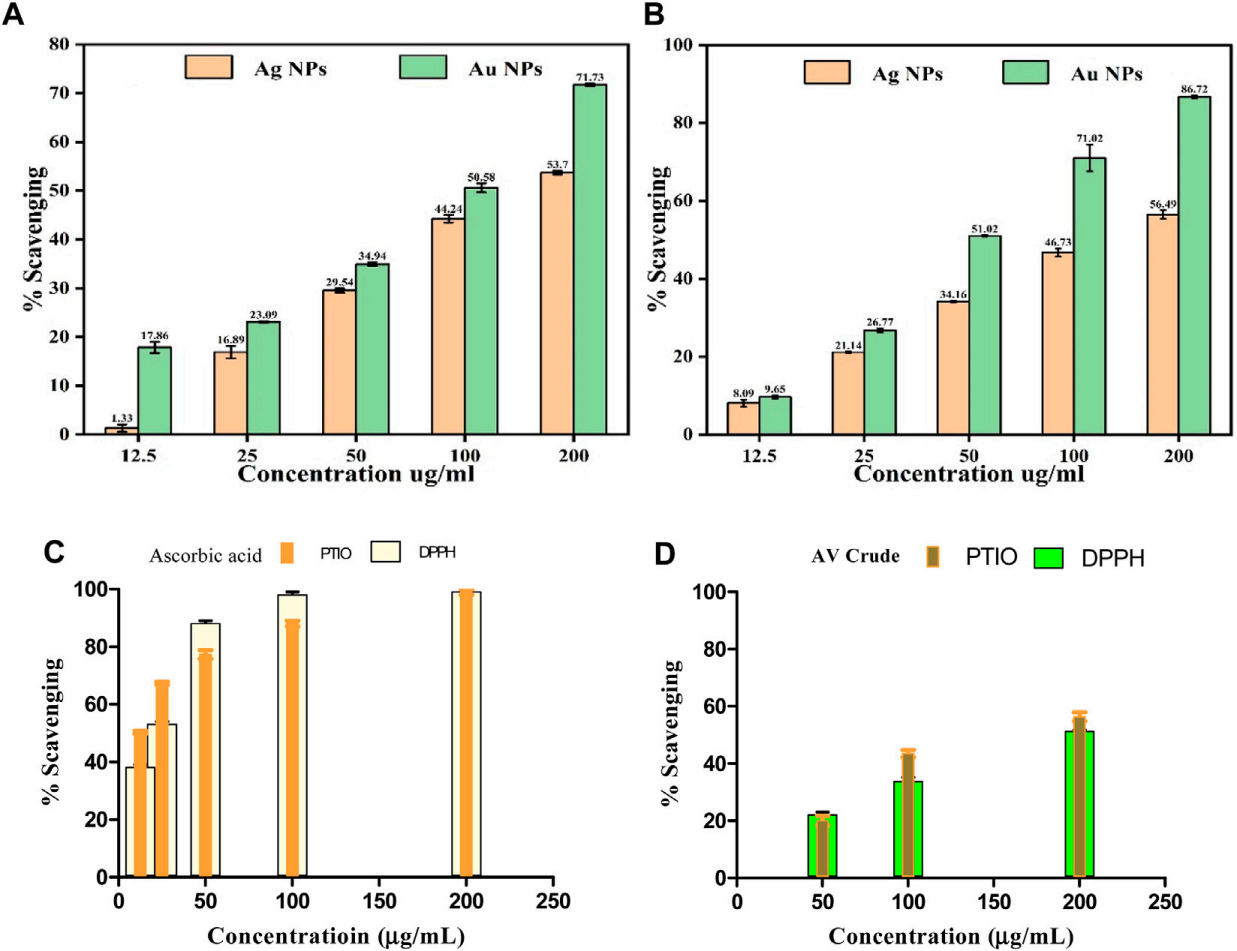
Antioxidant activity of AuNPs@AV and AgNPs@AV using **(A)** DPPH radical scavenging assay and **(B)** PTIO radical scavenging assay. **(C)** Ascorbic acid standard. **(D)** AV Crude.

Antioxidants play a vital function in safeguarding cells and tissues against oxidative stress, which arises from free radicals within the body. These free radicals are exceedingly reactive molecules that have the potential to impair DNA, proteins, and lipids, giving rise to diverse health complications. AuNPs@AV and AgNPs@AV were assessed for their antioxidant potential using the widely employed 2,2-diphenyl-1-picrylhydrazyl (DPPH) scavenging activity assay (Sreelekha et al., 2021). Figure 8A presents the antioxidant activity of AuNPs@AV and AgNPs@AV at various concentrations. The findings unveiled that AuNPs@AV showcased a higher scavenging activity in contrast to AgNPs@AV. Specifically, at a concentration of 200 μg/mL, AuNPs@AV demonstrated an impressive scavenging activity of 71.73%, whereas AgNPs@AV exhibited a scavenging activity of 53.7% under the same concentration. Lower concentrations (100, 50, 25, and 12.5 μg/mL) of the NPs exhibited 50.58, 39.94, 23.09, and 17.86% scavenging activity for AuNPs@AV, and 44.24, 29.54, 16.89, and 1.33% scavenging activity for AgNPs@AV. Both NP demonstrated a clear dose-dependent effect. A quantitative assessment of their effectiveness is given by the half-maximal inhibitory concentration (IC_50_), which signifies the concentration of a substance needed to impede 50% of the oxidative response. Here, the IC_50_ value for AuNPs@AV was determined as 114.03 μg/mL, while for AgNPs@AV, it was 161.20 μg/mL. These calculated IC_50_ values underscored the robust scavenging capability of AuNPs@AV, as they notably fall below the values documented in existing literature (Srinithya et al., 2016; Surapaneni et al., 2018; Valsalam et al., 2019; Artiukh et al., 2022). The DPPH % activities for ascorbic acid and AV crude are presented in Figures 8C, D.

### 3.5 PTIO radical scavenging capability of AuNPs@AV and AgNPs@AV

The potential antioxidant effectiveness of green synthesized AuNPs@AV and AgNPs@AV was further evaluated using the 2-Phenyl-4,4,5,5-tetramethylimidazoline-1-oxyl 3-Oxide (PTIO) radical scavenging assay. PTIO is a stable, hydrophilic oxygen-centered radical exhibiting an unpaired electron located on the O atom, and an amine oxide zwitterion moiety which makes it a hydrophilic species (Sabir et al., 2016). In this research, varying concentrations of 12.5, 25, 50, 100, and 200 μg/mL were employed for both green-synthesized AuNPs@AV and AgNPs@AV. The outcomes displayed in Figure 8B illustrate that the green-synthesized AuNPs@AV exhibited superior capabilities in scavenging ROS-derived free radicals compared to the green-synthesized AgNPs@AV.

At the minimal concentration of 12.5 μg/mL, the synthesized AuNPs@AV and AgNPs@AV exhibited inhibition rates of 9.09% ± 0.93% and 9.65% ± 0.45%, respectively. However, as the NP concentrations were raised to 200 μg/mL, the inhibitory activity significantly increased, reaching approximately 56.49% ± 1.1% for Ag and 86.72% ± 0.38% for Au. These findings demonstrated dose-dependent effects of the AuNPs@AV and AgNPs@AV. The standard used was ascorbic acid which showed maximum effect above 100 μg/mL in both free radical assays (IC_50_ = ∼23.2 μg/mL (DPPH) and ∼11.3 μg/mL (PTIO)) respectively. The crude showed a maximum 52%–58% activities, lower than the rest. Further study is still required to understand the mechanism of Ag and Au NP’s antioxidant effects and its potential applications in numerous health frameworks.

The IC_50_ values of AuNPs@AV and AgNPs@AV synthesized using environmentally friendly methods demonstrate that they are meaningful antioxidants. Concerning the IC_50_ determination, the green-synthesized AuNPs@AV and AgNPs@AV exhibited values of 149 ± 0.98 μg/mL and 80.05 ± 0.99 μg/mL, respectively. The AuNPs@AV demonstrate a lower IC_50_ value, signifying their high efficiency in inhibiting or scavenging the intended activity. Even at low concentrations, the AuNPs@AV exhibit a significant impact. This can be attributed to their intrinsic characteristics, including a large surface area, small dimensions, and potential interactions with biological compounds.

### 3.6 Photocatalytic performance of AuNPs@AV and AgNPs@AV

**FIGURE 9:**
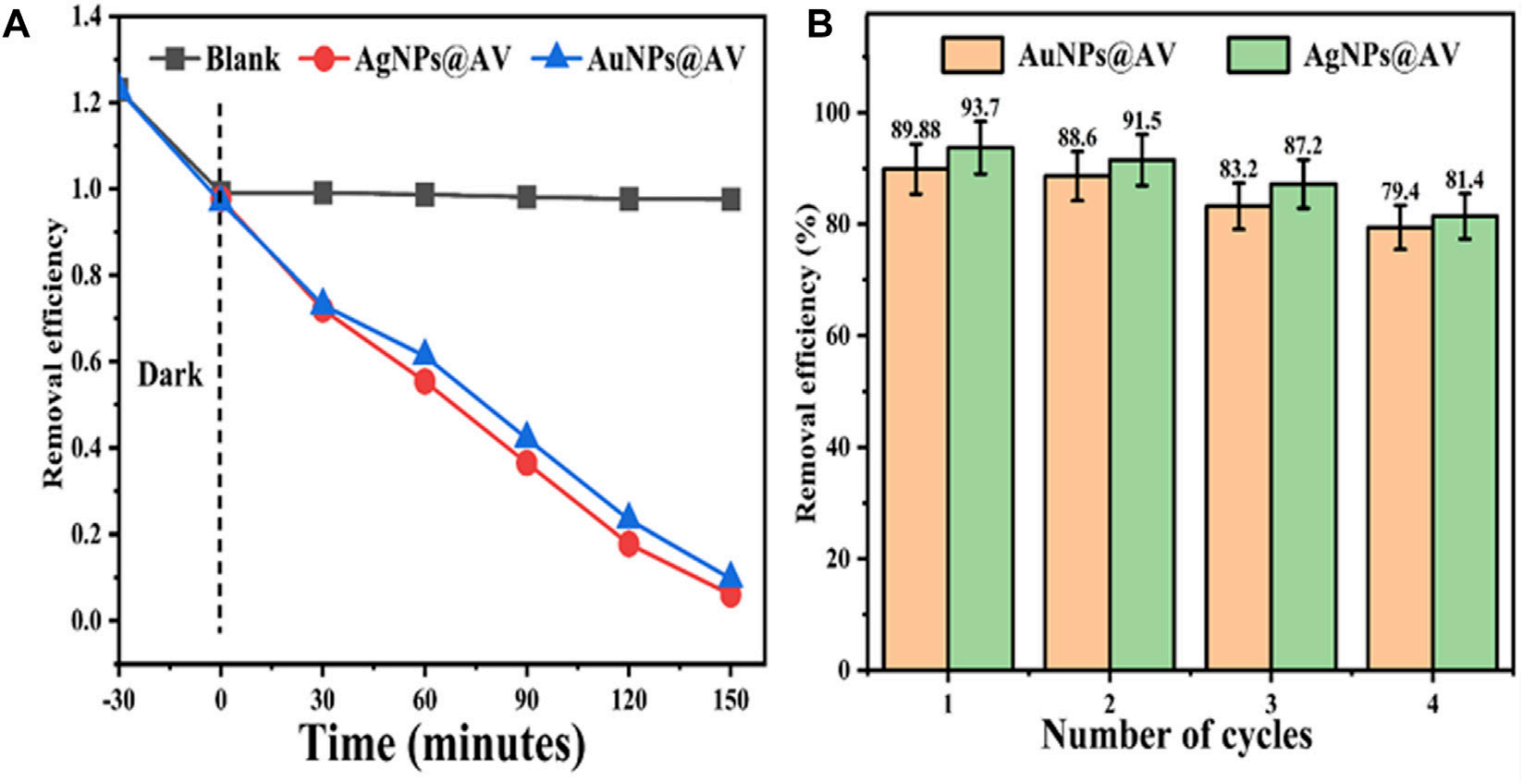
Photocatalytic degradation of methylene blue using AuNPs@AV and AgNPs@AV **(A)** and their recyclability **(B)**.

Green synthesized metals NPs has also demonstrated photocatalytic activity towards pollutants decontamination (Chandraker et al., 2019b; Chandraker et al., 2021b; Saied et al., 2022). The AuNPs@AV and AgNPs@AV were assayed for photocatalytic degradation of MB dye to investigate their photocatalytic efficiency. Figure 9A represents the % degradation of MB dye photodegradation at various irradiation times. Both types of AuNPs@AV and AgNPs@AV showed high catalytic activity and removed 89.88% and 93.7% methylene blue from the system respectively at room temperature. Furthermore, the NP-based photocatalysts were processed for recycling experiments to investigate their stability. Figure 9B represents the sustainability of both the photocatalysts. Both types of NPs show high stability till the fourth cycle where, for AuNPs@AV dye was removed 89.88% in the first cycle and 88.6, 83.2% and 79.4% in the second, third and fourth cycles respectively. Similarly, for AgNPs@AV, the efficiency in the first cycle was 93.7 and 91.5, 87.2% and 81.4% in the second, third and fourth cycles respectively.

### 3.7 Phytochemical studies

The AV crude extract was also screened for its natural constituents using simple phytochemical tests. The results showed the presence of alkaloids and flavonoids in good amounts while terpenoids and saponins were present in lesser amounts (Table 3). Alkaloids and flavonoids are known as good reducing and capping agents of Ag/Au precursor ions in solutions (Chugh et al., 2021).

**TABLE 3:** Presence of phytochemicals in crude extract of *Aconitum violaceum*.

S. No	Class of natural products	Test used	Presence
1	Alkaloids	Wagner’s test	+++
2	Flavonoids	FeCl_3_ test	+++
3	Terpenoids	H_2_SO_4_ coloration test	+
4	Saponins	Froth formation test	++
5	Anthrquinones	HCl/NH_3_ test	+

Key, +++ = appreciable amounts; ++ = Moderate; + = very less.

## 4 Conclusion

Green synthesis has been a proven effective and sustainable approach towards fabricating of metal NPs for biological and environmental applications. This study summaries an eco-environment friendly method in obtaining spherical and monodispersed gold and silver nanoparticles using *A. violaceum* crude extract having alkaloidal and flavonoid contents, acting as capping agents. Both AuNPs and AgNPs were characterized through different analysis which provided an insight to their morphology, functionalities, surface behavior and crystallinities. Both materials were effective antibacterial, antioxidant and potent in photo degradation of Methylene Blue, a pullutent under UV light within 150 min. The proposed strategy has the potential for wider implementation in biomedical research and environmental remediation.

## Data Availability

The raw data supporting the conclusion of this article will be made available by the authors, without undue reservation.
